# Utilization of gasification slag and petrochemical incineration fly ash for glass ceramic production

**DOI:** 10.3389/fchem.2022.1095500

**Published:** 2023-01-12

**Authors:** Zhenyu Hao, Hai Zhang, Xiaoli Tang, Lihua Sui, Yanan Li, Shucai Zhang

**Affiliations:** State Key Laboratory of Safety and Control for Chemicals, SINOPEC Research Institute of Safety Engineering Co, Ltd., Qingdao, China

**Keywords:** glass ceramics, Fe_2_O_3_, crystallization kinetics, PIFA, TCLP

## Abstract

This study investigated glass ceramics produced using coal gasification slag (CGS) and petrochemical incineration fly ash (PIFA) to immobilize hazardous heavy metals such as Cr and As. However, the crystallization kinetics and stabilization behavior mechanism of different heavy metals in the petrochemical incineration fly ash-derived glass-ceramics remains unclear. And X-ray diffraction, differential scanning calorimetry, scanning electron microscopy, and inductively coupled plasma mass spectrometry were used to characterize glass and crystalline products. In this paper, we reported the crystallization kinetics and chemical leaching characteristics of the glass ceramic. A low crystallization activation energy of 121.49 kJ/mol was achieved from crystallization peak of several different heating rates around 850°C, implying that it is easier to produce the glass ceramics at that temperature. The Avrami parameter of the former crystallization was determined to be 1.23 ± .12, which indicated two-dimensional crystal growth with heterogeneous nucleation. The toxicity characteristic leaching procedure results indicated that the heavy metals were well solidified, and that the leaching concentration was significantly lower than the limit specified by governmental agencies. The potentially toxic element index of the parent glass and the two glass ceramics were 11.7, 5.8, and 3.6, respectively. Therefore, the conversion of hazardous petrochemical incineration fly ash and other solid waste into environmentally friendly glass ceramics shows considerable potential and reliability.

## 1 Introduction

Oil accounts for one third of the global energy consumption and is the largest share of all the energy categories (Dutta et al., 2019; Melichar and Atems 2019; Dong et al., 2020). The rapidly developing petrochemical industry in China is expected to make China the largest producer of refined oil and ethylene by 2022 (Fang et al., 2021). The disposal of solid residues from various industrial thermal processes, such as petrochemical incineration fly ash (PIFA), has increasingly become a concern. PIFA, which contains multiple and large amounts of potentially risky heavy metals, is regarded as hazardous waste in the National Hazardous Waste List (2021 edition). Therefore, the treatment and disposal of PIFA and bottom slag has recently become an important social and environmental issue (Nikravan et al., 2018). Therefore, the effective recycling of hazardous wastes from the petrochemical industry has become an urgent requirement to realize the waste-free city goal in China (Fu et al., 2014).

While crude oil extraction and natural gas production have modernized our society, the environmental pollution cost has been more severe than expected (Blumer and Sass 1972; Almeda et al., 2014; Beyer et al., 2016; Ghorbani and Behzadan 2021). Incineration is one of the most common, effective and high-performance technologies for the disposal of municipal solid waste (Liu et al., 2019; Zhao et al., 2019), oil sludge (Gong et al., 2018), oil shale ash (Konist et al., 2020), and medical wastes (Thind et al., 2021). However, the disposal of fly and bottom ash produced by the incineration of various types of waste results in the occupation of large amounts of land resources (Allegrini et al., 2014), which is accompanied by secondary pollution problems, such as the transfer of heavy metals to the gas phase (Li et al., 2003; Tian et al., 2012). Although coal gasification technology as an environmentally friendly method has become an important part of the energy strategy in China (Wei et al., 2018), it creates large amounts of by-products.

Glass ceramics have the dual advantages of glasses and ceramics, such as environmental friendliness and excellent mechanical properties. Glass ceramics are polycrystalline materials produced by the melting/quenching of raw materials. The current mostly used common technology for treating hazardous solid waste is the heavy metal stabilization mechanism, which is realized by bonding the hazardous solid waste into the glass matrix, substituting the elements in the crystal phase with the hazardous solid waste, and forming a new crystal compound (Guo et al., 2017).

Thus, effectively recycling waste materials into a variety of ceramic products could be a promising strategy to solve this problem (Karpukhina et al., 2014). Previous studies reported that various solid wastes, such as coal fly ash (Albertini et al., 2013), metallurgical slags (Deng et al., 2020; Ceylan et al., 2021; Shang et al., 2021), and oil shale fly ash (Luan et al., 2010), have been used as part of the raw materials to produce glass ceramics. To the best of our knowledge, studies on the use of coal gasification slag and PIFA in glass ceramic matrices are limited. However, the use of pure chemical reagents and nucleating agents increases the cost of waste-based glass ceramics. Therefore, this study uses solid waste to realize all-waste-based fly ash glass ceramics, which is of great significance for sustainable social and economic development.

The main purpose of our research is to use PIFA and gasification slag as the main raw materials for the preparation of glass ceramics, and to prepare samples based on all-waste-based fly ash glass ceramics. The transformation and characteristics of the raw materials and glass-ceramics were analyzed using X-ray fluorescence (XRF) spectroscopy, X-ray diffraction (XRD), and scanning electron microscopy (SEM). The main goals of this study are 1) to investigate the crystallization kinetics, such as thermal stability, crystallization activation energy, and crystallization index, of these materials; 2) to establish a theoretical and technical foundation for developing an environmentally friendly glass ceramic by combining hazardous waste; and 3) to demonstrate the leaching behavior and potential environmental risks of the glass ceramics using different heat-treatment processes.

## 2 Materials and methods

### 2.1 Materials and reagents

The PIFA used in this study was acquired from a hazardous waste incinerator in southern China. The gasification slags used in this study were acquired from a refinery in Shandong Province, China. The PIFA and gasification slag samples were crushed and sieved to a size <.125 mm using a ball grinding mill (FRITSCH, Pulverisette 7, Germany). The collected samples were dried in an oven at 105°C for 24 h before the analyses. The chemical compositions of the samples were determined using XRF (ZSX Priums, RIGAKU, Japan), and the results are presented in Table 1. Acetic acid, hydroxylammonium chloride, ammonium acetate and hydrochloric acid used in this study were purchased from Sigma-Aldrich (Shanghai, China) and were used as received.

**TABLE 1:** Chemical composition of the coal gasification slag and petrochemical incineration fly ash (wt%).

	SiO_2_	K_2_O	Na_2_O	CaO	MgO	Al_2_O_3_	Fe_2_O_3_	MnO	P_2_O_5_	TiO_2_	LOI
PIFA	.36	.48	55.40	.31	.10	.03	1.43	.02	.27	.07	41.53
Slag	41.39	2.05	1.05	12.59	.72	12.86	11.28	.14	.12	.55	17.25

### 2.2 Characterization analyses

**FIGURE 1:**
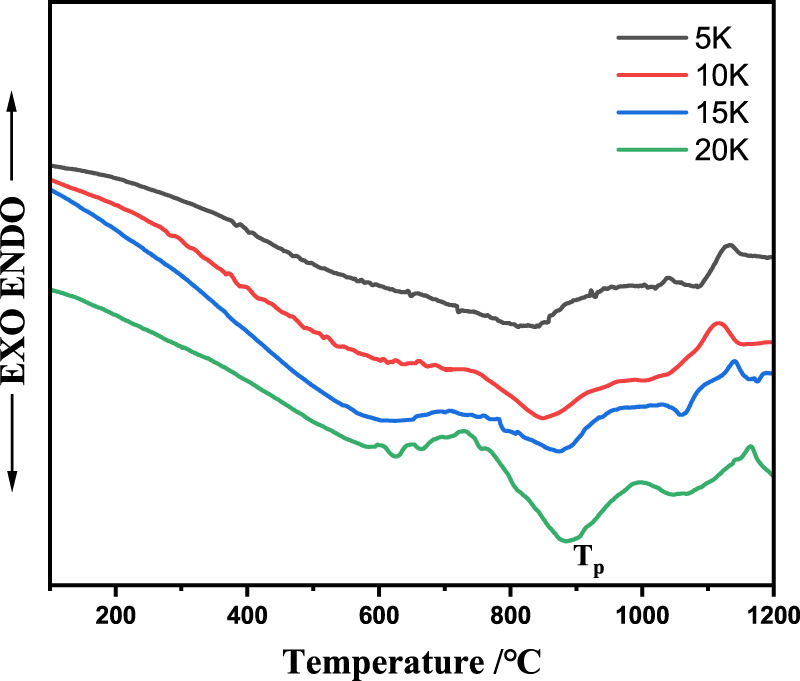
The DSC curves of the fly ash-slag-based parent-glass samples with different heating rate ranging from 100°C to 1,000°C/min: 5°C/min, 10°C/min, 15°C/min, and 20°C/min.

The thermal behavior of the basic glass sample was analyzed from room temperature to 1,200°C using differential scanning calorimetry (DSC; STA 449F3, NETZSCH, Germany) at the heating rates of 5, 10, 15, and 20°C/min in a N_2_ atmosphere. To study the crystallization kinetics of the parent glass, DSC curve analysis are performed, and the details can be found in Figure 1. The temperature of the exothermic peak in the DSC curves is defined as the crystallization temperature, T_p_, which is often selected as the optimum nucleation temperature. The crystalline phases of the samples were investigated using XRD (Miniflex 600, Rigaku, Japan). The 2θ degree range was 5°-80° and the step angle was 2°/min under Cu Kα radiation at 40 kV and 30 mA. The crystalline phases were determined by comparing the peak intensities and positions with those of the International Centre for Diffraction Data (ICDD PDF-2 Release 2004). And using the Rietveld refinement method to assess the validity of the XRD data. The morphology of the glass ceramics was characterized using a high-resolution SEM (MIRA LMS, TESCAN, Czech Republic).

### 2.3 Preparation of glass-ceramics

Glass samples were prepared from PIFA and gasification slag with a mass ratio of PIFA:gasification slag of 20:80. In each batch, the mixed samples were melted in a platinum crucible for 2 h in an electrically heated furnace at 1,300°C to ensure complete melting. The melt was then immediately quenched into water to obtain glass frit. The water-quenched glass was then dried in an oven at 105°C for 4 h, followed by grinding and screening (200 mesh) for use. The treated powder was placed in a cylindrical mold (*Φ* 19.05 mm) and pressed at 40 MPa using a powder compressing machine (YLJ-40TA, Hefei Kejing Materials Technology Co., Ltd., China). The mold containing the sample was then placed in a muffle furnace and heated to the nucleation temperature at 5°C/min and maintained at this temperature for 1h, followed by further heating to the crystallization temperature (850°C and 1,050°C) for 1 h or 2 h and the details can be found in Figure 1. The glass ceramics were obtained after naturally cooling to room temperature.

### 2.4 Heavy metal determination and leaching test

The total and leaching concentrations of heavy metals in the PIFA and glass ceramic samples were analyzed using inductively coupled plasma mass spectrometry (ICP-MS; Agilent 7800, United States). The safety and stability of the samples were estimated through leaching experiments using the toxicity characteristic leaching procedure (TCLP). The leaching fluid used was an acetic acid solution with pH 2.9. Then, 1.0 g of crushed sample and 20 mL of extraction fluid were mixed in a leaching vial and adequately rotated for 16 h. The state of the heavy metals in the glass samples were analyzed using a modified four-step sequential extraction procedure based on a method reported in the literature (Rauret et al., 1999). Table 2 shows the detailed processes.

**TABLE 2:** Detailed parameters of the Modified Sequential Extraction Method for based on GB/T 25282-2010.

Step	Concentration	Agent	Time (h)	Speciation
F1	40 mL, 1 mol/L	CH_3_COOH	16	Mild acid-soluble
F2	40 mL, 1 mol/L	NH_2_OH⋅HCl	16	Reducible
F3	10 mL, 30%	H_2_O_2_	1	Oxidizable
	50 mL, 1 mol/L	CH_3_COONH_4_	16	
F4	15 mL	HCl⋅HNO_3_⋅HF⋅HClO_4_	3	Residual

### 2.5 Environmental risk assessment

The synthesis toxicity index model (STIM) was established to explore the changes in the environmental toxicity of targeted heavy metals (Luan et al., 2009). The potentially toxic elements (PTEs) index used for the environmental risk assessment was calculated using Eq. 1 (Devi and Saroha 2014).

$$PTEs=\overset{m}{\underset{i=1}{\sum }}T_{i}\left(\frac{\overset{m}{\underset{j=1}{\sum }}\left(E_{j}Q_{i}^{j}\right)}{C_{N}^{i}}\right)$$ (1)

where *n* is the number of heavy metal species; *m* is the number of chemical speciation (*m* = 4); *T*_*i*_ is the toxicity response coefficient of heavy metal *i*; *E*_*j*_ is the bioavailability of chemical speciation *j* of heavy metal *i*; $Q_{j}^{i}$ is the content of chemical speciation *j* and $C_{n}^{i}$ is the background value of *i* in the natural environment (Hakanson, 1980). For a more comprehensive and reliable assessment, the background values refer to the secondary living standard of GB-15618-2008 (Luan et al., 2018).

## 3 Results and discussion

### 3.1 Characterization of the raw materials

Table 1 lists the chemical compositions of the PIFA and gasification slag. SiO_2_, CaO, Na_2_O, Al_2_O_3_, and Fe_2_O_3_, which are essential for glass-ceramic preparation, accounted for more than 80% of the raw material. Additionally, the Fe_2_O_3_ content in the gasification slag was approximately 12.86%. To the best of our knowledge, Fe_2_O_3_ is a commonly used nucleating agent in the glass industry (Alizadeh et al., 2004; Kang et al., 2019; Chen et al., 2021). The chemical composition of PIFA contained 40% Na_2_O and gasification slag contained SiO_2_, CaO, Al_2_O_3_, and Fe_2_O_3_ was suitable for implementation in the precursor glass raw material. Consequently, the SiO_2_-CaO-Al_2_O_3_-Na_2_O-Fe_2_O_3_ (fly ash: gasification slag = 20:80%) glass-ceramics were designed for the experiments. The sintering method was used to prepare solid waste glass ceramics with a higher crystallinity.

### 3.2 Crystallization kinetics

Thermal analysis has been used to study the crystallization kinetics of various glass systems. Figure 1 shows that the DSC curves of the prepared base glasses, which were analyzed at different heating rates (*α* = 5, 10, 15, and 20°C/min) to determine the crystallization activation energy. The DSC curves of the base glass exhibited two peaks at approximately 850°C and 1,050°C. The crystallization peak temperatures (*T*_*P*_), with respect to the increasing heating rate (β °C/min), were 827.6, 854.8, 878.4, and 886.6°C, respectively. The second crystallization peak for the different heating rates was at approximately 1,050°C. The crystallization exothermic peak temperatures of the base glass at the heating rates of 20°C and 5°C/min were 886°C and 827°C, respectively. The *T*_*p*_ of the base glasses with the same chemical composition gradually increased and the crystallization peaks at different temperatures increased and broadened with a continuously increasing heating rate. The base glass did not have sufficient nucleation time with an increasing heating rate, and crystallization became relatively delayed. When the heating rate increased, the hysteresis was more severe and the crystallization temperature was further increased.

**FIGURE 2:**
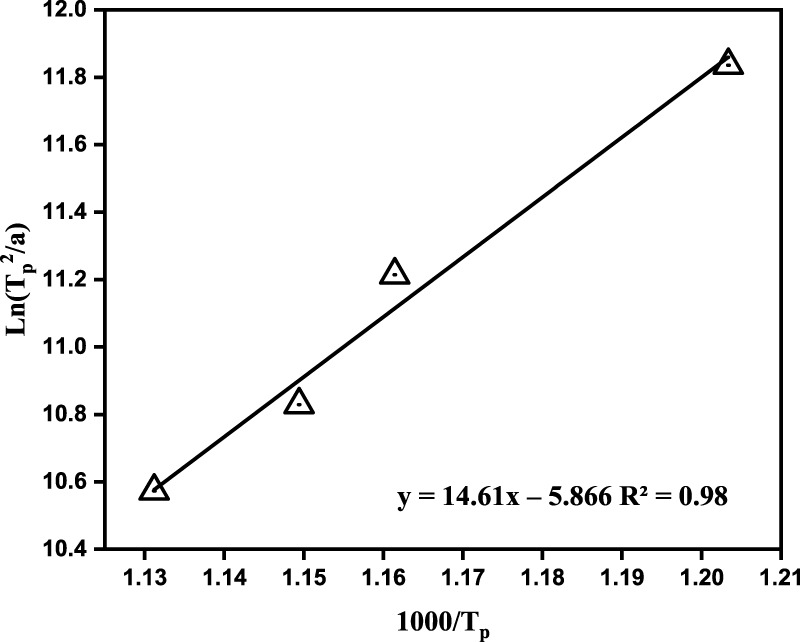
Linear fitting results of ln (T_p_^2^/α) ∼1,000/T_p_, *R* ^2^ is the Pearson correlation coefficient.

The crystallization kinetics can be determined using the Kissinger (Kissinger 1956) and Augis-Bennett equations (Augis and Bennett 1978), which are defined in Eqs 2, 3, respectively. Therefore, Eqs 2, 3 were adopted in this study to investigate the crystallization activation energy (*E*_*c*_) and the crystallization index (*n*) of the gasification slag-based parent glass.

$$\ln\left(\frac{T_{p}^{2}}{\alpha }\right)=\frac{E_{c}}{RT_{p}}+\ln\left(\frac{E_{c}}{R}\right)-\ln\vartheta$$ (2)

where $E_{c}$ (kJ/mol) is the crystallization activation energy; *α* (°C/min) is the DSC heating rate; $T_{p}$ (°C) is the temperature of the exothermic peak in the DSC curve as shown in Figure 1; R is the gas constant per mole [8.314 J/(K mol)]; and *ν* is the frequency factor. After calculating *E*_*c*_, the crystal growth index (*n*) can be obtained using the Augis-Bennett equation, as defined in Eq. 3.

$$n=\frac{2.5T_{p}^{2}}{{∆T}_{f}E_{c}/R}$$ (3)

where *ΔT*_*f*_ (°C) is the full width at half maximum of the exothermic crystallization peak shown in Figure 1; ‘*E*_*c*_ */R*’ is the slope in Figure 2 and $T_{p}$ (°C) is the temperature of the exothermic peak in the DSC curve.

Figure 2 shows the relationship between ln(*T*_*P*_^2^/α) and 1,000/*T*_*P*_. As previously mentioned, the *Ec* must be obtained before calculating *n*. The *Ec* of the gasification slag-based glass ceramic was calculated from the slope in Figure 2 as 121.49 kJ/mol. A glass melt requires a certain activation energy to overcome the energy barrier when it transforms from a high-energy glassy state to a crystalline state. For example, the *E*_*c*_ of blast furnace slag-based glass ceramics, fused-cast basalt crystallization, HCFS-based glass ceramics, and pickling sludge are 300–400, 238, approximately 200, and 187 kJ/mol (Bai et al., 2016a; Bai et al., 2016b; Zhao et al., 2020). The low *E*_*c*_ of the gasification slag and PIFA indicated that the parent glass crystallized more easily. To our best knowledge, the addition of Fe_2_O_3_ greatly reduces the precipitation temperature of the relevant crystals, resulting in a decreased *E*_*c*_ (Wang 2010). In this study, an *E*_*c*_ of only 121.49 kJ/mol was required to form the glass ceramics for the base glass. However, it is worth noting that although the *E*_*c*_ was lower than that of other waste-based samples, the glass ceramics prepared in this study could result in a decrease in the mechanical properties of the final product. Relevant studies have shown that Fe_2_O_3_ has little effect on the crystal phase type but has a greater effect on its crystallinity and precipitation temperature (Kang et al., 2019). Therefore, both the Fe_2_O_3_ content and the heat treatment temperature and time greatly influence crystal precipitation, which in turn affects the properties of the obtained glass ceramics. Therefore, the improvement of the mechanical properties of glass ceramics should be studied further.

*n* represents the number of growth directions as well as the nucleation and crystal growth mechanism. When *n* is greater than 3, the parent crystal is a bulk crystal, which is a three-dimensional volume-dominated crystal, and when n is 0–3, the crystal is a surface crystal, which is a two-dimensional surface-dominated crystal (Hosono and Abe, 1992; Hosono and Abe, 1994). In this study, the mean value of n was 1.23 ± .12, and the obtained *n* was not an integer, indicating that crystallization occurred through more than one mechanism. Because the gasification slag contained 16.17% carbon residue, the formation of the base glass and the crystallization kinetics were studied in an oxidizing atmosphere, in which iron will be oxidized to Fe^3+^. Karamonov *et al.* found that an oxidizing atmosphere could lead to a decrease in *n* (Karamanov et al., 2000). As previously mentioned, due to the presence of Fe^3+^ as the main form of iron in this system, the content of Fe_2_O_3_ in the formulation system was 9.3% and acted as the main nucleating agent, resulting in two-dimensional surface-dominated crystallization, which resulted in a lower Avrami parameter (Kim and Park 2020).

### 3.3 Crystalline phase analysis

XRD analysis was performed on the thermally treated glass ceramics at 850°C and 1,050°C to identify the crystalline phase. The NaO-CaO-Al_2_O_3_-SiO_2_-Fe_2_O_3_-based glass is the basic silicate system that is widely used in many industrial fields and solid waste recycling, in which Fe_2_O_3_ can effectively promote the nucleation rate as a nucleating agent (Erol et al., 2007).

**FIGURE 3:**
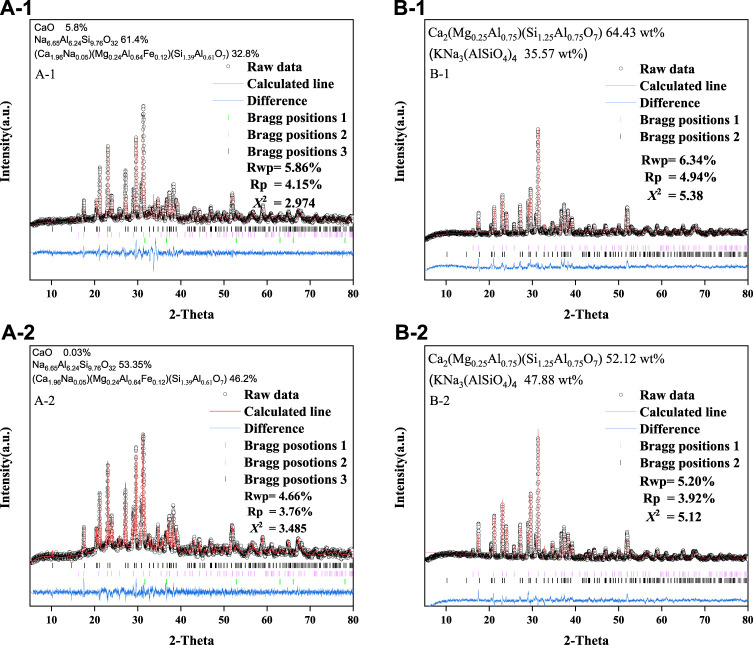
Rietveld refinement analysis of the XRD patterns for A-1 and B-1 at 850°C, and A-2 and B-2 at 1,050°C. For A-1 and A-2: Bragg position 1 is CaO; Bragg position 2 is KNa_3_(AlSiO_4_)_4_; Bragg position 3 is Ca_2_(Mg_0.25_AI_0.75_) (Si_1.25_AI_0.75_O_7_). For B-1 and B-2: Bragg position 1 is Na_6.65_Al_6.24_Si_9.76_O_32_; Bragg position 2 is (Ca_1.9_6Na_0.05_) (Mg_0.24_Al_0.64_Fe_0.12_) (Si_1.39_Al_0.61_O_7_).

Figure 3 shows the XRD patterns of the base glass after heat treatment. After crystallization at 850°C for 1 and 2 h, the main crystalline phases were (Ca_1.96_Na_0.05_) (Mg_0.24_Al_0.64_Fe_0.12_) (Si_1.39_Al_0.61_O_7_) (PDF No. 72-2128), nepheline (Na_6.65_Al_6.24_Si_9.76_O_32_) (PDF No. 83-2372) and calcium oxide (CaO) (PDF No. 99-0070). When the crystallization temperature was raised to 1,050°C, the main crystalline phases were forsterite Ca_2_(Mg_0.25_AI_0.75_) (Si_1.25_AI_0.75_O_7_) (PDF No. 79-2422) and nepheline KNa_3_(AlSiO_4_)_4_, (PDF No. 74-0387). Clearly, the crystallization time had no significant effect on the formation of the phases in this study, and the XRD peak of the 2 h treatment was slightly higher than that of the 1 h treatment. In summary, toxic elements may be incorporated in the crystalline phase or fixed within the glassy matrix during this process.

**FIGURE 4:**
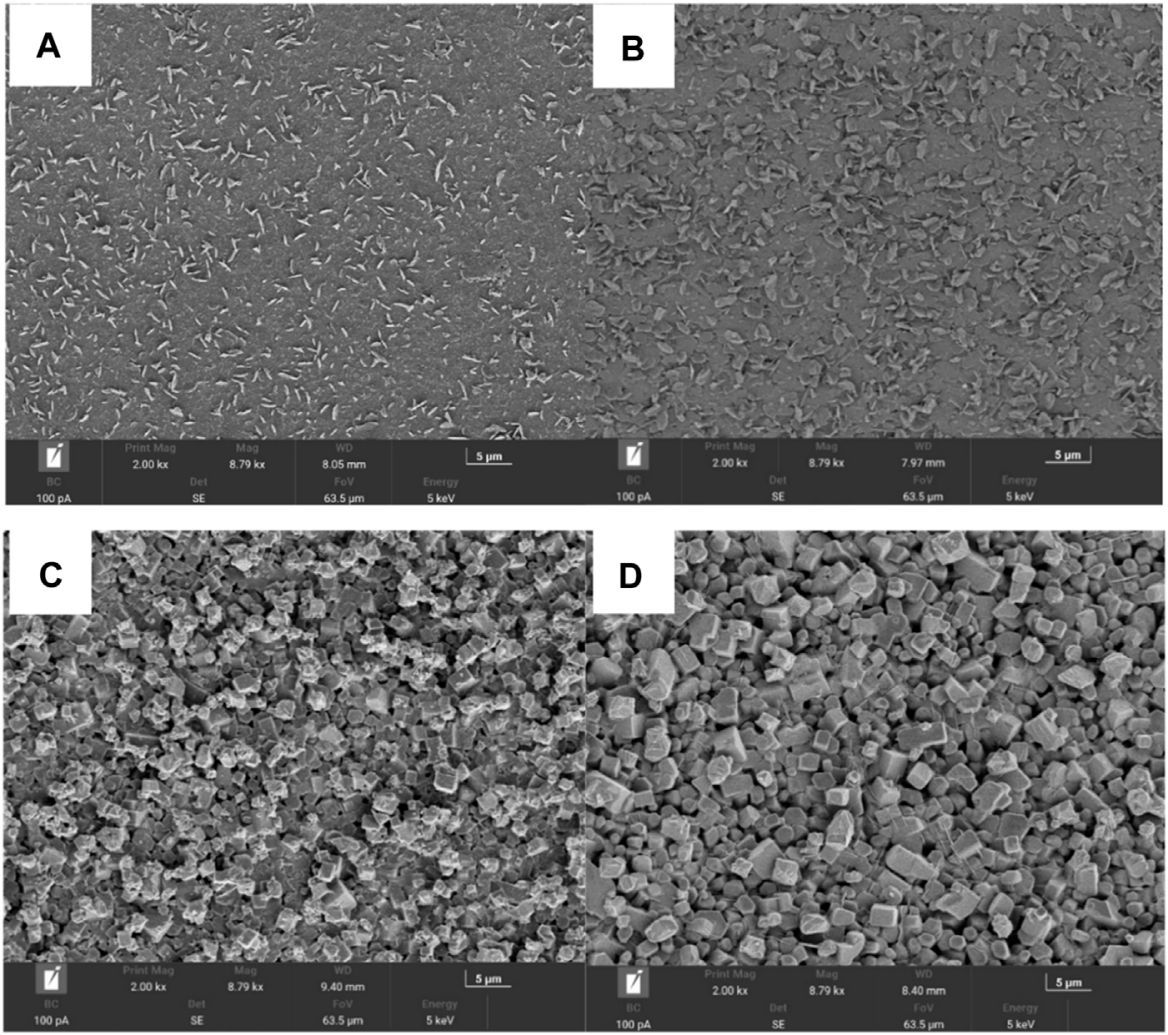
The SEM images of gasification slag-based glass ceramics prepared by **(A)** at 850°C for 1 h, **(B)** at 850°C for 2 h, **(C)** at 1,050°C for 1 h, and **(D)** at 1,050°C for 2 h.

Figure 4 shows the surface morphology of the glass ceramic microstructure of the particles. The particle size of the prepared glass ceramic samples was approximately 1 μm and the arrangement was relatively uniform. Figures 4A, B depicts the microstructure of the glass ceramics crystallized at 850°C, which shows some oriented disordered platelets and a large number of crystals distributed in the glass matrix. With an increasing crystallization time, thinner and larger multi-layer flaky crystals with a disordered orientation were observed in the glass ceramics treated for 2 h. Figures 4C, D shows that in the glass samples continually sintered at 1,050°C for 1 and 2 h, the flaky crystals disappeared and some short columnar crystals were formed. When the heating time increased to 2 h, the boundary of the crystallized grains was clearer. Additionally, the glass ceramic crystals, which exhibited a higher degree of crystallization and a more uniform grain distribution, became larger and denser at higher crystallization temperatures. Furthermore, the crystals exhibited a particle stacking arrangement. However, the base glass was not only precipitated as a single material crystal during the heat treatment process, but also had a variety of secondary crystal phases, mainly because there were many impurities in the slag and PIFA, which influence the crystal precipitation process.

### 3.4 Leaching tests

The total heavy metal concentrations in all the gasification slag and PIFA glass ceramics were determined using ICP-MS, which are listed in Table 3. The leaching concentrations of chromium and arsenic in the PIFA were significantly higher than the limits specified in GB5083.3-2007 and GB16889-2008. The leaching concentrations of chromium, arsenic, and zinc were 269.8, 158, and 43.5 mg/L, respectively. The leaching concentrations of heavy metals in the slag did not exceed the limits specified in GB5083.3-2007 and GB16889-2008. Thus, the PIFA used in this study cannot be disposed in a landfill without further treatment. Previous studies have shown that waste PIFA easily reacts with heavy metals at high temperatures to form low-boiling heavy metal chlorides (Zhang et al., 2020). In this study, the volatilization of heavy metals during heat treatment were negligible, as chloride was not detected.

**TABLE 3:** Total concentration and leaching concentration of heavy metal contents of the PIFA and gasification slag.

		V	Cr	Ni	Cu	Zn	Cd	Ba	Pb	Mn	As
Total concentration (ppm)	PIFA	2244	2811	108	37.5	672	.063	8.14	3.13	260	683.4
	Slag	82.3	53.4	29.6	35.0	43.8	ND	4152	27.6	1210	43.8
Leaching concentration (ppm)	PIFA	ND	269.8	.08	1.4	43.5	.04	.24	.12	ND	158
	Slag	ND	.01	.01	.012	.016	ND	.184	.05	ND	.019
	Limit level^a^	—	5	5	100	100	1	100	5	—	5

^a^ GB 5085.3 Identification standard for hazardous waste-identification for extraction toxicity.

ND, not detected.

### 3.5 The solidification of the parent glass and glass ceramic

**FIGURE 5:**
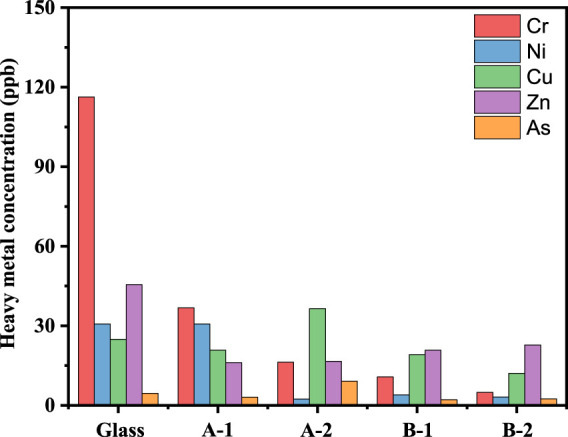
Leaching concentration of Cr, Ni, Cu, Zn and As in different sample. A-1: sintered at 850°C for 1 h, A-1: sintered at 850°C for 2 h, B-1: sintered at 1,050°C for 1 h, B-2 is sintered at 1,050°C for 2 h.

Figure 5 shows the TCLP leaching concentrations of the existing heavy metals in the different glass ceramics of gasification slag co-processed with PIFA. The leaching concentrations of chromium, nickel, copper, zinc, and arsenic of the glass and glass ceramic samples complied with the requirements of the United States EPA and the Chinese national standards.

After the first and second heat treatment, the leaching heavy metal concentrations in both samples (A-1, A-2, B-1, and B-2) showed a significant decreasing trend, where the decreasing trend of chromium was the most significant, decreasing from 116.3 to below 30 ng/g^−1^. However, the leaching concentration of arsenic remained in the steady state level for the different crystallization times or heat treatments. This is because acetic acid was used as the buffer solution in the TCLP leaching experiments, which hardly reacts with the crystallite phase but can react with the glass phase. Increasing the sintering temperature is beneficial for reducing the leaching concentration of heavy metals in the samples, mainly because metal ions participate in the phase transition and intercalate into the crystal structure of the glass ceramics at high temperature. In conclusion, the preparation of glass ceramics can be used as an effective method for PIFA solidification and stabilization.

### 3.6 Potential ecological risk assessment of heavy metals

**FIGURE 6:**
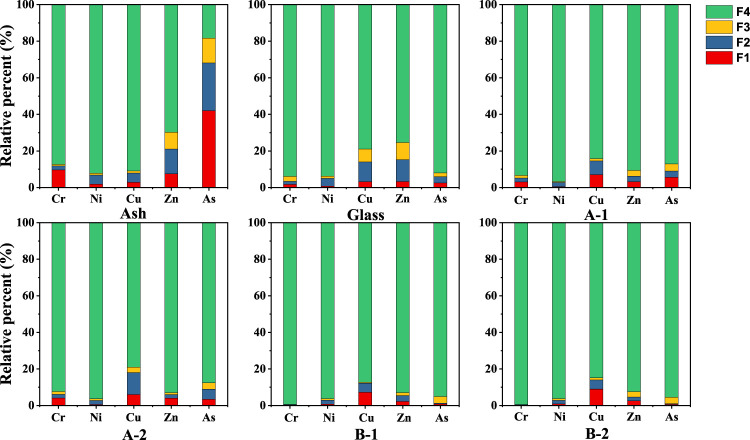
Chemical speciation distributions of heavy metals in glass and ceramics-glass. A-1: sintered at 850°C for 1 h, A-2: sintered at 850°C for 2 h, B-1: sintered at 1,050°C for 1 h, B-2 is sintered at 1,050°C for 2 h.

Heavy metals can exist in glass ceramics in the acid-soluble, reduction, oxidation, and residual states. The distribution of the four forms of heavy metals in different solids strongly affects their leaching behavior and potential toxicity risk to the environment. Figure 6 shows the distribution of the different heavy metal chemical forms in the eutectic solidified body.

Nickel, copper, and zinc exist in PIFA as the residual state; therefore, the leaching concentration is low, as shown in Figure 6. However, the residual state of chromium accounts for 9.6% of the total existent states. It is worth noting that the acid-soluble state of arsenic is up to 40% in the chemical speciation distributions. This indicates that the toxicity of PIFA mainly originates from chromium and arsenic. The oxidizable and reducible states of chromium and arsenic in the glass matrix was <4% and 6%, respectively. The main form of the five heavy metals in the glass ceramic samples A-1 and A-2 was the residual state, all of which were >75%. The predominant form of chromium and arsenic after secondary crystallization at 1,050°C was the residual state, which increased significantly to 99% and 95%, respectively. There was no discernible difference in the nickel and zinc forms between the parent glass and glass ceramic. Measurement of the weight of the solid matter before and after the reaction indicated that the vitrification process resulted in a lower mass loss rate of 5%–10%, which was mainly due to the decomposition of carbonate in the system. The organic fly ash was measured using ion chromatography, and no chloride ions were detected. Therefore, at high temperature, zinc will not react to generate heavy metal chlorides. Additionally, heavy metals form more stable and insoluble species (such as metal and mineral salts), resulting in poor leaching capacity (Guo et al., 2017), which can indicate that the glass ceramic has a high heavy metal fixation efficiency. The contribution of the different heavy metals was similar at different temperatures, and the slight difference is likely due to experimental errors.

**FIGURE 7:**
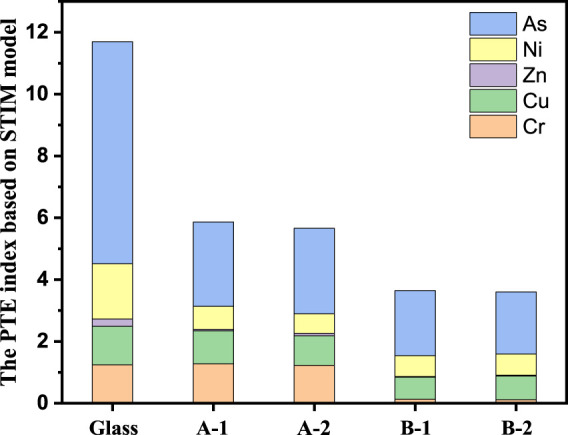
PTEs index of heavy metals. A-1: sintered at 850°C for 1 h, A-1: sintered at 850°C for 2 h, B-1: sintered at 1,050°C for 1 h, B-2 is sintered at 1,050°C for 2 h.

The STIM assessment method can comprehensively reflect the potential impact of heavy metals on the ecological environment. According to STIM, the synthesis toxicity index (STI) value of each sample can be calculated using Eq. 1, which is equal to the PTEs index, as shown in Figure 7. The base glass had a toxicity value of 11.7. Arsenic is the main elements responsible for toxicity, which of PTEs index is 7.2. The PTEs indices of the glass ceramics sintered at A: 850 and B: 1,050°C was <5.8 and 3.6, respectively. The PTEs index of the glass ceramic was lower than that of the parent glass. Glass ceramic, as compared to glass, reduces the potential risk of heavy metals polluting the ecological environment. Based on PTEs calculations and residential land soil background values, a PTEs of 0–132 indicates a mild level (Luan et al., 2018), which means that the glass ceramics obtained in this study can be used as building materials for their high value utilization.

## 4 Conclusion

The crystallization kinetics of the glass ceramics prepared from coal gasification slag and PIFA were studied. The thermal stability of the PIFA-based parent glass was reported first. The samples were prepared with a fly ash: slag ratio of 2:8 and were treated with four heating rates (*α* = 5, 10, 15, and 20°C/min). The low crystallization activation energy (121.49 kJ/mol) implied that it is easier to make glass ceramics. The Avrami parameter of the former was determined to be 1.23 ± .12, indicating the formation of two-dimensional crystal growth with heterogeneous nucleation at 850°C. These results indicated that using gasification slag and PIFA to produce glass ceramics both facilitated their recyclability and saved energy. At 850°C, the main crystalline phases were (Ca_1.96_Na_0.05_) (Mg_0.24_Al_0.64_Fe_0.12_) (Si_1.39_Al_0.61_O_7_), Na_6.65_Al_6.24_Si_9.76_O_32_, and CaO. When the crystallization temperature increased to 1,050°C, the main crystalline phases were Ca_2_(Mg_0.25_AI_0.75_) (Si_1.25_AI_0.75_O_7_) and KNa_3_(AlSiO_4_)_4_. The glass ceramics, as compared to the parent glass, enhanced the solidification efficiency of heavy metals. The distribution of the four forms of heavy metals, their leaching behavior, and potential toxicity risk to the environment were investigated. The base glass exhibited a PTEs value of 11.7 and the glass ceramic sintered at 850°C and 1,050°C exhibited PTEs values of 5.8 and 3.6, respectively, indicating that the risk of environmental pollution was greatly reduced. This reduced risk was mainly due to the heavy metals existing in the glass ceramics in the form of residues. From the sustainable development and low environmental risk perspectives, using PIFA and other silicate solid waste to produce glass ceramics is a potential and promising technique for the solidification/stabilization of heavy metals. However, further studies on improving the mechanical and physical properties of the mixed materials are required for the practicability and environmental benefits of slag and ash recycling.

## Data Availability

The original contributions presented in the study are included in the article/supplementary material, further inquiries can be directed to the corresponding author.

## Abbreviations

XRF, X-ray fluorescence; XRD, X-ray diffraction; SEM, scanning electron microscopy; PIFA, petrochemical incineration fly ash; TCLP, toxicity characteristic leaching procedure; PTE, potentially toxic element; STIM, synthesis toxicity index model; STI, synthesis toxicity index; DSC, differential scanning calorimetry; ICDD, the International Centre for Diffraction Data; PEI, potential ecological risk.
